# Severe Anti-HMG-CoAR Necrotizing Autoimmune Myopathy Secondary to Statin Use

**DOI:** 10.1155/2022/6120424

**Published:** 2022-09-16

**Authors:** Priyanjali Pulipati, Jayakar Kamantha Reddy, Syed Ali Husain

**Affiliations:** St Joseph Mercy Oakland, 44405 Woodward Ave, Pontiac, MI 48341, USA

## Abstract

Immune-mediated necrotizing myopathy is an uncommon but debilitating disease that can be triggered by drugs, toxins, or cancer. It is similar to polymyositis in presentation but is differentiated by findings on muscle biopsy. We present a case of a 79-year-old male on statin therapy who presented with proximal muscle weakness and elevated creatinine kinase (CK) levels. He had a confirmatory muscle biopsy for immune-mediated necrotizing myopathy. Unfortunately, the patient's condition eventually escalated, involving respiratory and esophageal muscles in spite of prompt diagnosis and treatment.

## 1. Introduction

Immune-mediated necrotizing myopathy (IMNM) or necrotizing autoimmune myopathy (NAM) is a rare muscle disorder that is seen in about 21 people per million. It has a female predominance, with a usual age of onset of 30–70 years [1]. The etiology of IMNM includes toxins, drugs like statins, thyroid disease, cancer, and viral infections. It presents as acute or subacute proximal muscle weakness with elevated creatinine kinase (CK) and may progress to involve respiratory and cardiac muscles. There is autoimmune necrosis of muscle, with minimal or no inflammatory infiltrates seen on the muscle biopsy. IMNM can be associated with antisignal recognition particles (anti-SRP) and anti-3-hydroxy-3-methylglutaryl-coenzyme A reductase (anti-HMG-CoAR), with 20–30% seronegative cases [1]. Treatment depends on understanding the etiology and treating the underlying cause. Pharmacological therapy with corticosteroids, intravenous immunoglobulin (IVIG), or steroid-sparing agents is also beneficial [1]. The prognosis is worse than with inflammatory myositis, with 50% of patients having muscle weakness after 2 years of therapy [2], although more than 95% of the patients are alive 5 years after the diagnosis [3]. Follow-up is crucial with CK levels and physical examinations to determine muscle strength.

## 2. Case Presentation

A 79-year-old male with a history of diabetes mellitus type 2, hyperlipidemia, hypertension, reflux disorder, and recently diagnosed tumid lupus came into the emergency department with complaints of bilateral hip weakness, which led to a fall from a standing height. He recently underwent a skin biopsy with his dermatologist due to a rash over the torso and a positive ANA (1 : 320) and was diagnosed with tumid lupus (a cutaneous form of lupus) and initiated on hydroxychloroquine 200 mg twice daily and a tapering dose of steroids. A physical examination was remarkable for extensive areas of nonindurated erythematous to brown plaques over the upper body and decreased muscle bulk with bilateral hip and shoulder muscle strengths of 3/5. Laboratory tests were remarkable for creatinine 2.31, glomerular filtration rate 27, white count of 12,100, neutrophilic and normocytic anemia with hemoglobin 12.3, creatinine kinase was found to be elevated to 27,151, and sedimentation rate 102. Urinalysis shows 2+ protein, 3+ blood, and >50 RBCs. Hydroxychloroquine (HCQ) and his home medication, atorvastatin, were held with the initial suspicion of HCQ-induced rhabdomyolysis. It was deemed less likely due to steroid use as CK was elevated. High-dose steroids, with prednisone 60 mg daily, and aggressive fluid resuscitation were initiated. Rheumatology and nephrology were consulted. A renal biopsy was carried out due to suspicion of lupus nephritis, which showed acute tubular necrosis with diffuse foot process effacement compatible with minimal change disease and hypertensive sclerosis, ruling out lupus nephritis. Additional workup by rheumatology showed anti-SRP negative, anti-HMG-CoAR positive, anti-Ro positive, and aldolase 130, all suspicious for IMNM. The patient underwent a muscle biopsy of the thigh, which showed necrotizing myopathy without a significant lymphocytic infiltrate. A PET scan was performed to rule out malignancies. The patient continued to feel weak, and a pulse steroid dose of methylprednisolone 1 g/day for 3 days and a 5-day course of IVIG 0.4 g/kg/day were attempted with minimal change in clinical symptoms. He continued to deteriorate with progressive dysphagia and hypoxia, eventually requiring intubation followed by a tracheostomy and percutaneous endoscopic gastrotomy for about 6 months. During those 6 months, he was treated with 5 runs of plasmapheresis followed by 1 g/kg every 2 weeks and rituximab with an inadequate response. He is currently on maintenance with prednisone 30 mg daily and mycophenolate mofetil 1000 mg twice daily and is post reversal of tracheostomy and gastrostomy but is functionally quadriplegic and bedbound.

## 3. Discussion

IMNM is commonly anti-HMG-CoAR positive or anti-SRP positive or seronegative. Moderate to severe debility was found to be more common with anti-HMG-CoAR when compared to anti-SRP (71% vs 60%) [4].

HMG CoAR is an enzyme involved in cholesterol production. Autoantibodies against HMG CoAR can precipitate statin-induced necrotizing myopathy in patients using a statin. Although statin use is common, the incidence of IMNM in patients treated with statins is 2–3 cases per 100,000 patients [5], with a predominance in women older e than 50 years of age [6]. Among the statins, atorvastatin may be strongly associated with anti-HMG-CoAR IMNM compared to simvastatin and rosuvastatin [2]. Our patient described above was on atorvastatin 80 mg daily for hyperlipidemia before his diagnosis with IMNM. It is also interesting to note that nonstatin-triggered IMNM due to anti-HMG-CoAR antibodies can be found [7]. Class II HLA-DRB1*∗*11 : 01 and HLA-DRB1*∗*07 : 01 alleles are associated with anti-HMG-CoAR antibody production [8]. Statin-associated IMNM is associated with higher rates of dysphagia (46% vs 17%), but less often in men (45% vs 66%) when compared to statin-naïve IMNM [4].

An anti-SRP-positive IMNM is common in younger women in the fourth decade of life. These patients are more prone to cardiac, esophageal, and respiratory complications such as cardiomyopathy, dysphagia, and interstitial lung disease (10–20% in anti-SRP vs 5% in anti-HMG-CoAR [7]) [2]. The prognosis is poor, with only half of the patients regaining baseline strength after 4 years of treatment [2].

It is also crucial to differentiate IMNM from polymyositis (PM). Both conditions present with gradual or sudden and progressive proximal muscle weakness. PM is common in women around 20 years of age. PM is an inflammatory process and is distinguished from IMNM by an inflammatory infiltrate in muscle fibers on biopsy when compared to the necrosis and degeneration with minimal inflammation seen with IMNM.

The diagnosis of IMNM is confirmed by muscle biopsy. Electromyography and MRI may also assist in the diagnosis by differentiating neurologic etiologies for muscle weakness. Additional screening for cancers may be necessary. Pulmonary function tests or computed tomography of the chest are useful when interstitial lung disease is suspected.

Treatment of IMNM is based on clinical judgment, and no guidelines are available. High-dose steroids with oral prednisone (1 mg/kg/day) [2] or pulse intravenous methylprednisolone (1 g/day for 3–5 days) are usually recommended [9]. Steroid-sparing agents (SSA) such as methotrexate, rituximab, mycophenolate mofetil, and IVIG are also promising therapies. A recent study highlights triple therapy with SSA, IVIG, and steroids as the most efficient treatment induction strategy (4 out of 22; 18%, failed this regimen) [10]. They also proved the efficacy of IVIG and SSA by demonstrating that the corticosteroid-free regimens (i.e., IVIG or SSA) had achieved steroid-free remission in 100% (vs 73% in steroid-based), normal strength in 93% (vs 68% in steroid-based), and successful maintenance with SSA in 50% (vs 54% in steroid-based regimen) of the patients. A prompt diagnosis and treatment are crucial in IMNM since patients may develop dystrophic features due to fatty replacement of muscle [2].

## 4. Conclusion

Anti-HMG-CoAR IMNM due to statin use is rare but can leave devastating debilities, like in our patient. It is important to promptly identify and treat IMNM to try and limit the disability. It is yet to be understood if the occurrence of IMNM is formulation or dose-dependent on the statin. The gold standard for diagnosis is a muscle biopsy. Treatment guidelines for IMNM have to be elucidated, but currently available therapies include steroids, IVIG, and steroid-sparing agents like rituximab and mycophenolate mofetil.

## Data Availability

The data can be made available upon request from the corresponding author.
